# Supplementary material for: An information theoretic method to resolve millisecond-scale spike timing precision in a comprehensive motor program
Source: PLoS Comput Biol. 2023 Jun 12;19(6):e1011170. doi: 10.1371/journal.pcbi.1011170 (PMC10289674; doi:10.1371/journal.pcbi.1011170)
Supplement: S1 Text — Discussion of the reasoning for and underlying algorithms behind three separate methods to estimate spike timing precision from mutual information corrupted by uniform noise. Details on differences between methods and comparison between methods are discussed. (PDF) [file pcbi.1011170.s008.pdf]

Supporting Information for Ortega, *et al.* "An information theoretic method to resolve millisecond-scale spike timing precision in a comprehensive motor program"

S1 Text: Procedure for estimation of spike timing precision using three separate algorithms.

As the continuous precision estimation method of this paper requires selecting a noise level at which MI begins to drop, observed precision may be sensitive to the specific algorithm used to find the point of MI loss. To evaluate this sensitivity and choose an algorithm with favorable characteristics, we compared three different algorithms on the MI vs. noise amplitude curves for datasets with fixed, known precision levels (S5D Fig through S5F Fig) and on the main dataset of this paper (S5G Fig). The three algorithms were chosen as plausible but mechanistically distinct methods to find the noise level at which MI begins to fall. The "STD method", used in the main text, finds the noise level where MI drops below the lower bound on the estimate of MI at zero noise (defined as mean MI minus one standard deviation). The "derivative method" instead estimates precision level as the noise level where the 2nd derivative of I_C w.r.t. r_c peaks, akin to peak downward acceleration of the MI estimate. Differentiation was performed with a 2nd order Savitsky-Golay filter with an 11-sample window, and peak finding was subject to a minimum amplitude requirement and prominence requirement. The "Line intersection method" treats the MI vs. r_c curve similar to a phase transition, fitting two lines to the approximately linear low-noise and high-noise regions and deeming precision as the noise level where these two lines intersect. Lines were fit with orthogonal regression to the first and last 30 noise levels.

All three algorithms were run on simulated and real datasets with precision fixed to known levels, as in Fig 5. On this data of known precision, the STD threshold and derivative methods had similarly consistent variance in precision estimation, both outperforming the line intersection method on consistency and overall estimation error. While the STD method displays a slight tendency to estimate higher values of precision for actual precision levels of 1ms, it demonstrated less bias than the derivative method, which consistently estimated precision lower than the actual level, with greater underestimation when the actual precision was higher. The STD method was chosen for the results in the main text of this paper based on its lack of bias and relatively consistent estimation error, but all three methods produced very similar results. When run on the main dataset of this paper, all three methods produced highly similar results, with no methods results different enough to change the interpretations and conclusions of this paper. The derivative method differed significantly from the STD method in its estimates of the precision for the AX and BA muscles, providing a lower mean precision near 1ms as compared to the mean precision from the STD method for both closer to 2ms. The line intersection method deviated significantly from the STD method for the SA, DVM, and DLM, estimating all three to have precision greater than 1ms (but never greater than 2ms). Despite their very different underpinnings, then, all three methods produced highly similar results which indicate a level of robustness to this method of precision estimation that is mildly agnostic to the exact method of selecting the noise level at which MI drops.
