# Supplementary material for: An information theoretic method to resolve millisecond-scale spike timing precision in a comprehensive motor program
Source: PLoS Comput Biol. 2023 Jun 12;19(6):e1011170. doi: 10.1371/journal.pcbi.1011170 (PMC10289674; doi:10.1371/journal.pcbi.1011170)
Supplement: S2 Text — Discussion of the potential effects uniform noise could have on the nearest neighbor distance distributions of an underlying dataset in the process of estimating spike timing precision. Potential issues related to noise corruption and the choice of k, and their relevance to the precision estimation method, are detailed and evaluated. (PDF) [file pcbi.1011170.s009.pdf]

Supporting Information for Ortega, *et al.* “An information theoretic method to resolve millisecond-scale spike timing precision in a comprehensive motor program”

S2 Text: Effect of noise and choice of k on nearest neighbor distances and the scale underlying mutual information estimation

The KSG estimator is built on the notion that the entropy of continuous variables can be estimated from a variable's density distribution, approximated from the average distance to the kth-nearest neighbor. In practice the estimator is more complex, but chiefly relies on max-norm distance to the kth-nearest neighbor to define the scale at which it operates. For the uniform noise corruption method of this paper, then, it is important to understand how the addition of uniform noise to spike times alters the kth nearest neighbor distributions underlying the mutual information estimation in Kraskov's algorithm.

S6 Fig shows the $k = 4^{th}$ nearest neighbor distance distributions in the $|z - z'|$ space used by Kraskov's algorithm for an example moth and muscle combination (S6A Fig and S6B Fig) and the entire dataset of this paper (S6C Fig and S6D Fig), calculated according to Kraskov’s method. Note that, as shown in S6C Fig and S6D Fig, the addition of uniform noise does shift the max norm $k = 4^{th}$ nearest neighbor distance to be greater, with the magnitude of the shift increasing with the number of spikes in a wingstroke (lighter colors, wing strokes with more spikes, have a greater shift). Such a shift in the max norm distance distributions could lead to difficulty in interpretation of precision values obtained from the continuous method, as the time scale at which precision can be estimated would be changing as the magnitude of uniform noise is increased. Rather than compare the same data with zero noise and with noise added at the same temporal resolution, MI may be estimated with a different temporal resolution for data with noise added, slightly complicating interpretation of precision.

These complications do not alter the conclusions of this paper, however, for several reasons. For one, the shift in the max norm k-NN distance distribution remains fairly minor, typically less than 1 ms even for spike counts of 5-7 spikes in a wing stroke. The drop in MI used to identify precision for all muscles occurs at noise levels well before those where median k-NN distances are shifted more than 0.5-1 ms from the zero noise case. For two, only extreme amplitudes of uniform noise raise the median k-NN distance above 1 ms, and only for high spike counts which actually contribute a very small amount to the total MI observed. The vast majority of all wing strokes are less than 4 spikes (see S6C Fig), and thus 80% or more of the overall estimate of MI for each muscle does not have max norm distance distributions shifted by much relative to the observed precision. The red dashed lines in S6C Fig of the max norm k-NN distance distribution medians, weighted by probability of that many spikes in a wing stroke in the same manner MI is calculated and averaged across moths, illustrates this. This adjusted k-NN distance shift *never* exceeds 0.3 ms and thus stays far below any identified precision level.

Note that while the shift in max norm k-NN distance distributions due to added uniform noise is not a problem for the dataset of this paper, it may pose a problem for datasets applying this method with fewer observations or much higher dimensionality. As seen in S6 Fig, k-NN distances shift more if the dimensionality of the data is higher: Since the estimator operates on a scale set by the *max*-norm distance, the probability of there being a difference between two spike

times that is increased by the addition of uniform noise to all spike times is greater the more spikes there are. For finite data, if a uniform noise of $r_c = 6$ ms is applied to only 2 spike times, it is less likely that the max-norm difference between spike times is increased the maximum possible amount of 6 ms than if the same noise was applied to 5 spike times. More spikes means more chances for the max norm distance to approach r_c. Further applications of this method to estimate spike timing precision in other datasets should be cautious to check that their input dimensionality and noise amplitude do not combine to greatly alter the max norm k-NN distance distributions with added noise.

Another key validation of the continuous precision estimation method, related to the temporal resolution Kraskov's MI estimator operates on, is the choice of k. In this paper, $k = 4$ was used for all main figures and analysis. But other values of k will lead to entropy estimation on different scales; while in the limit of infinite data the choice of k should not dramatically alter the MI estimated, in practice lower values of k lead to greater statistical error while higher values of k lead to greater systematic error. To ensure the estimation of precision is invariant to the exact scale of operation of the MI estimator, and that the choice of k does not alter the precision observed, we ran the continuous KSG estimator at $k = 2$ through $k = 7$ (S7 Fig). For all muscles, across all moths, the choice of k did not significantly alter the observed precision. Estimated precision values from the continuous method and related conclusions, then, are not sensitive to the choice of k for this dataset. This result is not general, however, so it is prudent for applications of this method to new datasets to perform a similar validation.
