# Supplementary material for: An information theoretic method to resolve millisecond-scale spike timing precision in a comprehensive motor program
Source: PLoS Comput Biol. 2023 Jun 12;19(6):e1011170. doi: 10.1371/journal.pcbi.1011170 (PMC10289674; doi:10.1371/journal.pcbi.1011170)
Supplement: Attachment — Submitted filename: PNS_PLoSCompBio_ResponseToReviewers Final.pdf [file pcbi.1011170.s010.pdf]

We thank all three reviewers for reading our manuscript and providing insightful comments. It has taken us some time to complete these revisions because the first author graduated and the senior author went on paternity. However, we are pleased to resubmit this manuscript after extensive revisions. Several of the comments from multiple reviewers pushed us to greatly expand our analyses around the comparison of the NSB and KSG methods. We think that these comments have greatly improved the manuscript. We start this response by highlighting the major changes that we have made:

1. We added simulated data with known precision so that we could assess how well the two methods perform in this known context.
2. We have added a much more extensive analysis of the sensitivity of our results to the choice of k nearest neighbors and have analyzed the kth nearest distances to assess how they impact our estimate of precision.
3. We have incorporated an estimate of bias into the NSB method.
4. We have introduced two new ways of establishing the threshold for precision in the datasets. In addition to our confidence interval method, we now include one based on breakpoint analysis and another based on finding the extremes in the second derivative of the data.
5. We have incorporated many of these changes into the main paper and now provide a supplement with many more details on some of these analyses, especially when they are primarily of relevance to the specialist. We maintain the overall organization and focus of the manuscript both in comparing the methods and using them to test an important biological hypothesis about the degree of precision across a comprehensive motor program.
6. We have substantially revised the text to incorporate many of the excellent inquiries and clarifications the reviewers requested and to discuss the changes above.
7. Much of the work in revision was done with a new graduate student in the lab, Leo Wood, who has now been added to the author list for his contributions.

Thank you for your time and consideration of our work.

Joy Putney, Tobias Neibur, Leo Wood, Rachel Conn, & Simon Sponberg

----

Reviewer #1: *In their manuscript "An information theoretic method to resolve millisecond-scale spike timing precision in a comprehensive motor program", Putney and colleagues develop a novel method for calculating the precision with which motor unit spikes encode motor force output by utilizing information theoretic tools. Putney and colleagues take advantage of a data set from their earlier publication in which they managed to record from the 5 major bilateral pairs of flight muscles of the Hawkmoth Manduca sexta during tethered flight, with the mutual information tools correlating the activity of the recorded muscles with output from a simultaneously recorded force/torque transducer attached to the tether. The authors develop a method to calculate mutual information between these two signals without resorting to temporal binning of either signal, leading to improved estimation characteristics for the calculated information rates. Finally, the authors corrupt the spiking signal by adding increasing amounts of uniform noise to the timing of the muscle spikes, and determine the temporal precision of the motor units as the added noise amount at which mutual information rates*

between the corrupted spike train and force output significantly deviate from mutual information rates between the original spike train and force output. The authors then compare the efficacy of their method with a traditional approach to estimating spike precision using tubal information estimates derived from temporal binning.
The manuscript is generally well-written and makes appropriate use of the unique advantages offered by using their model organism. However, the authors should address one major concern before the manuscript would be ready for publication.

> We thank the reviewer for their concise and accurate description of our manuscript. We especially appreciate the positive comments with respect to the significance of the work and the opportunity to response to the feedback below.

My major concern deals with the presentation and discussion surrounding figure 4 (and corresponding supplementary figures 2 & 3), which compares the authors' novel mutual information estimation with a traditional binning approach. As the authors are presenting their approach as an improvement over prior methods, I view this as the central figure of the manuscript, however some of the comparisons here felt underwhelming. First of all, in cases where the discrete method can produce an estimation of the temporal precision (e.g. figure 4A, Moth 3 R3AX from figure S2), the authors should present this number, perhaps showing the 2 region regression mentioned in the methods section in order to help the reader see difficulties with the estimation.

> We thank the reviewer for this suggestion; a more thorough and comprehensive comparison between the two methods was warranted. We've now included in Figure 4 a comparison of all the precision values obtained by both methods, as well as a new figure (Figure 5) where the two methods are evaluated on data with precision fixed to known ground-truth levels. Some of the advantages of the continuous method suggested here are principled ones (not having to assume a reduced number of states, the inherent limits to estimating discrete words of finer and finer precision, etc). However, as suggested by the review we have now added a more extensive empirical comparison to show that these differences do matter. We do find that the discrete method gives larger predictions for precision thresholds (less precise spiking) and this seems to be consistent with the idea that the discrete approach may necessarily underestimate the mutual information because of the limitations described. Please note that Fig. 4 has also been updated to include bias estimates for the discrete method which make the story clearer as well.

Second, the authors have not fully justified the method they use for determining the temporal precision from the mutual information curve (currently determined as the point at which the mean MI estimate for the noise added signal drops below the mean - SD of the noise-free signal. However, given the width of the SD several valid alternatives could give relatively different precision values (e.g. where the mean + SD of the noise added signal drops below the mean of the noise free signal). The authors should fully justify their choice here, or perhaps use a more standard approach such as T-Test at each noise level,

determining the precision as the point at which the two distributions of information estimates diverge at the 95% confidence level.

We thank the reviewer for suggesting an alternate method for choosing spike timing precision in each muscle. We implemented the suggested method of t-testing the distribution of 150 MI estimates at each noise level against a hypothesized mean of the zero-noise MI level, choosing precision as the noise level where the t-test rejected the hypothesized mean with a 95% CI. Unfortunately, this method does not seem to capture a reasonable fall-off point where the MI begins to rapidly drop with added noise amplitude, instead typically choosing the second or third lowest levels of noise as the precision point. We owe this mostly to the fact that the plateau region of the precision figures is not truly flat, with a very slow drop in MI that is detectable by a t-test when the distribution sample size is N=150.

To better justify our choice of method, we have now included two other alternative methods for determining the break point to establish precision. These are included in the supplemental figure S5 and we discuss a comparison of the resulting precision values for the entire dataset between the three different methods. Alongside the original mean - SD method, we include selecting precision as the negative peak in the 2nd derivative of MI with respect to log10(r_c), and a method similar to how phase transformations are discretized by finding the intersection of two orthogonal regressions fit to linear sections of the MI vs. log10(r_c) graph. Despite the stark differences in how these three algorithms find spike timing precision, on the same data all three methods produced very similar results, with differences in mean precision estimate for each muscle less than 1ms. Thanks for prompting us to do this because we think it strengthens the conclusions.

Along the lines of both the prior two points, the manuscript would greatly benefit from any metric which evaluates the difficulty in determining the precision from the mutual information curves. The cases shown in figures 4, S2, and S3 give anecdotal evidence along these lines, but for publication in a computational journal the authors should not shy away from quantifying this difficulty (in cases where precision can be calculated at all).

To allow for quantitative evaluation of both methods, we have now included a figure evaluating both the NSB and KSG based precision estimation methods on synthetic and real datasets where precision is fixed to known ground-truth levels (Figure 5). Because evaluating the performance of each precision estimator to ground truth provides an understanding of how easily and accurately they are able to assess precision, we think this addition should provide a satisfactory metric.

Additionally, to better give not just anecdotal cases but a more thorough coverage of examples, we have included supplemental figures S2 and S3 which show the MI curves for all muscles and moths, for both methods. Both the new supplemental figures and the precision estimator evaluation figure (figure 5) greatly benefited from the inclusion of shuffling bias correction to the NSB method, which actually allowed for estimation of precision on all muscles and moths. Because of this, this data is now also shown in the main text in the updated Figure 4 to allow better comparison between the KSG and NSB methods.

Finally, I would caution the authors against arguing that estimates yielding higher precision (i.e. smaller values of Rc/Rd) are necessarily better estimators, as the authors tend to do in comparing figure 4A with 4C. In this case we don't know the ground truth temporal precision. Instead the authors should stick with the strength of their method, which is the (numerous) cases in which a discrete estimator of mutual information can not produce an estimate of the temporal precision at all. I this vein I would recommend that the panels 4A and 4C are subbed out with panels from figures S2 and S3 to show a case in which there is not plateau at high bin sizes as discussed in line 239.

> We agree with the reviewer that caution must be used when we don't have a groundtruth. However based in part on this and other reviewers' comments we decided to actually test these methods on simulated data where we do have a groundtruth. This helps bolster our claims and understanding of both methods, indicating the biases and accuracy of both methods on datasets of different sizes and dimensionalities. Discussion of these groundtruth simulations has been added throughout the text, such as the section starting at L287. In short, the KSG method
>
> We have also added a more thorough discussion of why the NSB estimator often does not plateau at high numbers of bins, including a supplemental figure S4 which illustrates the undersampling bias at high bin count and motor output discretizations which occurs with NSB.

The panels in S3 should also be modified to show the MI error estimation at zero noise, as done with the purple shaded regions in figure 4C&D.

> We have added the purple shaded regions to S3 to make this figure consistent with 4C&D, and thank the reviewer for this good suggestion.

Minor comments:

Throughout the manuscript, the authors refer to "small" and "large" precision (e.g. lines 78, 260, 264, 344, 362). This goes against the conventional usage of "high" and "low" precision, and causes unnecessary confusion with other measured quantities, such as larger and smaller rates of mutual information. I highly recommend that the authors carefully go through the manuscript and uniformly refer to "high" precision (i.e. smaller values of Rc/Rd) and "low precision (i.e. larger values of Rc/Rd) in order to minimize confusion.

> Thanks! Good idea. We have corrected the examples given, and have also gone through the manuscript to ensure that our usage of "high" and "low" to describe precision is consistent throughout.

Figure 2: mention of the continuous distributions (blue) and the spike count distribution (black) during explanation of the discrete solution is uneccessarily complicated. I would recommend at least removing all mentions of the spike count distribution as it does not elsewhere figure into the manuscript, and if mentions of the continuous timing are kept then it should be noted that this does not relate to the discretized estimation being done throughout the rest of the figure. Also, the authors should check that the color of the figure is correct, as the quantities described as grey in the text appear more pink on my computer display.

We thank the reviewer for pointing this out. We have modified Figure 2 to remove the references to "Continuous" timing, with the blue text now described in the caption as the spike trains represented to sampling rate. Additionally, we had originally made one of the discretized distributions gray to represent the 1 bin case where the spike words were equivalent to a spike count code. We have changed the color scheme to match Figure 3, where the lowest precision spike word distribution is represented with dark pink (instead of gray) and the highest precision spike word distribution is represented in lighter pink, and removed references to spike count code in the figure. We have also edited the caption to reflect this change.

Line 208: "was assessed" should be "were assessed"

Line 404: "preservartion" should be "preservation"

Both corrected.

Reviewer #2: *The paper contains potentially very interesting and novel results about the precision of encoding of motor units. However, the paper is currently presented as a methodological information theoretic advance. However, the novelty of the methods of the paper is in part overstated and previous literature is not fully and not fairly acknowledged. Moreover, the new methodology is not fully rigorously tested on simulated data.*
I think that the paper should be improved before publication, and I offer my suggestions below. Once these issues are sorted, I think the paper would be a valuable addition to the literature.

The first problem I that the introduction of the paper seems to claim that all information theoretic methods, developed mainly for sensory systems, critically rely on the repetition of the same stimulus many times or on white noise, which is not true.

There are examples of information theoretic methods that computed the mutual information between a non-white-noise dynamical changing feature X (e.g. a feature of the external word such some feature of an image in a movie as in Ince Mazzoni et al J Neurosci Methods 2012, or of sound in speech as in Gross, Hoogenboom et al et al PLoS Biol 2013, or even a motor related signal as in Zuo and Diamond, Curr Biol 2018) and the time course of neural activity Y. These methods do not rely on the white noise assumption. Crucially, these methods do not need the repetition of the same stimuli many times in the exact way, and

in fact in some of the examples above the same methods were applied equally successfully to same stimuli repeated multiple times as in Ince et al or to stimulus repeated only once with a long naturalistic sequence as in Gross et al. Importantly, those methods see to me very similar in concept to the ones presented in this paper. Although the authors have a degree of novelty by adding the comparisons of different ways to compute information and jitter the spike times, the methods of the authors share part of the logic of earlier work that they did not acknowledge. If the authors feel that this opinion is mistaken and the logic of their method is very different from previous work of course they can argue so, but after acknowledging fairly the previous work.

Thus sentence such as "Some methods used in sensory systems do not translate well to motor systems since sensory systems can be studied using repeated stimuli or white noise analyses that are impossible to replicate in the decoding problem of motor systems" should be modified to cite and acknowledge previous related solution to this problem and being more specific about the novelty here, which is to test different information theoretic methodologies and spike times manipulation techniques.

We thank the reviewer for this insight which we very much agree with. We have updated the sentence accordingly to better reflect our meaning and better reflect the prior literature. The new sentence(s) on lines L31-34 is now:

"Some methods used in sensory systems do not translate well to motor systems since sensory systems can be studied using repeated or controlled stimuli, as even in well-defined tasks like target reaching, variation exists in how a motor task is realized \cite{Georgopoulos1982,Wu2014}. Here information theoretic methods show promise as they don't require repeated stimuli or white noise assumptions \cite{ince2012novel, gross2013speech}."

Second, the inference that the Kraskov method provides a better estimation of the NSB method is based on examining the real data results. While the arguments of the paper are reasonable, these should be corroborated by systematic simulations studies to validate them. This is particularly important because the authors decided to pitch this study as a Methods one. I think that the authors should provide simulations of EMG signals of the type examined here, and then make statistical models of spike sequences encode some of the EMG features (possibly to kind of match in terms of time scales and precisions the different types of spike timing encoding that they later find in the data) and then test the algorithms. Under which conditions (encoding time scales, data sample sizes, encoding noise levels etc) does one algorithm work better that the other.

Thank you for this suggestion. Based on this comment and others we took significant time to revise this manuscript and incorporate simulations that use known levels of precision. These revisions are incorporated into a new main text figure, Figure 5, which evaluates both methods on synthetic and real data with precision fixed to ground-truth known levels. Synthetic data was chosen to match similar scales as the spike data of this paper, and both datasets when fixed to known precision levels allowed for systematic evaluation of the errors and biases of each method. We did not explore every possible parameter, instead opting for real and simulated datasets of varying dimensionality and encoding noise levels but similar sample sizes to the original data set of the paper. We decided this level of analysis strikes a balance between

exploring simulated data and still evaluating a specific hypothesis about the level of precision in our real data set of interest. In conjunction with the a priori reasoning about the limitations of the discrete approach, we think this establishes a strong basis for our method.

Finally, the language is sometimes loose and does not take into account the precision of definitions of previous literature. Temporal encoding is defined too loosely "sensory and motor systems convey information through the precise timing of spikes, or temporal encoding". Previous literature has defined temporal coding as coding of temporal features by spike timing whereas temporal encoding is reserved for when the spike times encode non temporal features (e.g. color of an image) or when spike times encode information ant time scales significantly faster than the time variations of the variable to be encoded, see Theunissen and Miller J Comput Neurosci 1995 which is incorrectly cited in this paper, and Panzeri et al Trends Neurosci 2010.

This distinction is an important one, so this sentence at line L2 has been updated to make the more subtle point more clearly:

"Neurons in both sensory and motor systems perform temporal encoding, where information on signals is carried in the precise timing of spikes, on a faster timescale than the characteristic timescale of the signals themselves"

Additionally, we have made sure that our usage of the term "temporal encoding" throughout the paper matches the proper definition. For the sake of clarity between the two terms there is now no mention of temporal coding in the paper, as it constitutes a less relevant concept for this paper.

Reviewer #3: *The authors present an information-theoretic method of investigating the spike timing precision relevant to motor control using a nearest neighbour estimator on continuous values, and demonstrate this method on a data set recorded from tethered moths. They also compare this to a baseline established method using discrete/binned estimators.*

Overall, the method is interesting and relevant to the audience. Strengths are the targetting of the approach to very relevant questions, much of the experimental design, and the couching of the contribution in the literature.
Yet, I do have some serious misgivings about the extents to which conclusions can be drawn from the methods, which need to be addressed in order to make the claims robust. These are outlined in the following:

1. Precision as supported by estimators

*We must remember that all of these quantities are *estimates*, not absolutes. So the precision at which the information seems to peak is not necessarily *the* precision the channel operates at, but the precision at which we're no longer able to measure mutual information with our estimator, given the samples that we're working with. It may only appear to be an upper limit then due to such estimation issues, or indeed a more efficient estimator may find more information at all time scales and even*

identify a more coarse grained resolution as a limit to the precision at this higher value of information. I'm not noting this sense of caution in your paper, and it does underpin some of the following.

A. The most important way this plays in is how the KSG estimator functions. The estimator itself selects a scale for operation determined by the (max-norm) distance to the k-th nearest neighbour of each point; the estimator thus operates at a scale that is supported by the amount of data that you have (more samples leading to smaller scales). That's worth commenting on on its own - the estimator simply does not get below that scale. The statement at line 227 - "This method is only limited in its representation of the spike timings by the sampling rate" - isn't correct in this light, because the real limit is the identified resolution of the scale of the k nearest neighbours.

We thank the reviewer for their excellent and nuanced set of critiques and suggestions. The aforementioned statement and other similar statements throughout have been revised to better reflect how the KSG estimator functions. In part because of the mentioned limitations of the estimators, we decided to incorporate the simulations of Figure 5 which more thoroughly evaluate the ability of both estimators to measure precision. We have also made sure throughout the paper to more carefully and deliberately acknowledge the nature of the results as merely estimates, and the limitations inherent in these estimates. Some examples of this new wording:

L276-280: “Precision estimation with the continuous method is still limited; precision not only cannot be smaller than the minimum time representation of 0.1ms defined by the 10kHz sampling rate, but also the Kraskov *k*-nearest neighbors estimator operates at a resolution determined by the kth nearest neighbor distance, bounding the resolution of entropy estimation…”

L326-329: “With the continuous method, the KSG estimator operates at a scale which varies with the sample size and distribution of the data…”

B. More importantly, it would be important to explore how adding noise to your data starts to interfere with the selection of these k-th nearest neighbours, and the neighbours in the marginal spaces. If your noise is at a much smaller scale then it would not be expected to have an effect. As your noise increases, you're also likely to be increasing the k-th nearest neighbour scale to some degree because of destroying the correlations between the variables. I would suggest that you explore how the distributions of k-nn distances across samples in the estimates without noise (even just via their medians) relates to the identified precision limits. This would seem to be important to explain how the method is functioning.

This is a very important point, thank you for prompting us to explore it further. To better address this and check that adding noise does not significantly increase the scale of operation of the KSG estimator, we have added supplemental figure S6. As we cover in the new supplemental, while uniform noise of sufficient amplitude does shift the max-norm kth-nearest-neighbor distance distributions, this shift is pronounced only for high input dimensionality of 4 or more spikes per wing stroke, and does not shift the scale of operation (IE the nearest

neighbor distance) by more than 0.3ms at even the most extreme amplitudes of noise with the highest input dimensionality. We have also added more discussion of the nearest neighbor distance distributions and how scale of the KSG estimator functions in finding a scale of precision to the text to better connect these points and aid any readers. Some examples of this added discussion:

L207-212: “Our continuous estimate of MI…”

Paragraph at L257, starting with “Here, we present an extension of a continuous MI estimation method…”

C. Along similar lines, which value of k was used here? This isn't mentioned, but is important for repeatability. You note at line 277 - "For this data set, these checks had been done previously [13], and for any data set the stability of estimates would need to be assessed." I can see in fig 3 of that paper that the MI seems to be quite stable w.r.t. k up to 10, which is not unexpected for this estimator. However those investigations were without adding noise, and as outlined above, different k values will lead to different scales of resolution, and given that this is precisely what you're investigating here I would suggest that you explore how your identified precision limits change with k.

We have now added more mention throughout the text that k=4 was used (such as at L227, the caption to Figure 4, and L358), as well as a new supplemental figure S7 which addresses how estimated precision is affected by the choice of k. As is covered in this figure, there was no detectable change in precision from any value of k from 2 through 7, so we feel confident that our precision estimation is relatively robust to the choice of k. Further mention of this has been added to the text and a discussion of the importance of this check for future applications of this method have been included.

D. fig 5 and Line 372 "It could be that having multiple spikes to carry motor information decreases precision requirements for each spike" - these both indicate larger time scales being identified for the upper limit of precision for channels with larger spike rates. More spike times to consider means higher dimensional data for the KSG estimator, which usually means larger knn distances and therefore lower resolution for the same number of sample points. That seems to very naturally leads to the more coarse grained resolution which you identify as a result here, and so is a confound that needs to be investigated. I would suggest in the first place examining how the knn distances change w.r.t. spike rate and muscle types here, and how these then relate to the precision limits as above.

Thank you for prompting us to look into this. Higher dimensional data does indeed lead to both a greater likelihood of greater max-norm nearest neighbor distances even without added noise and a greater shift in the max-norm nearest neighbor distance distributions with added noise (as shown in supplemental figure S6). However, for the sample sizes and input dimensionalities explored in this paper, we found that this is not a major confound that alters the results presented.

When data is set to ground-truth precision levels in Figure 5, we see that the estimator estimates precision within ±0.5ms of the true precision level, even for muscles of high dimensionality and many spikes such as the AX and BA. This is the same data that conclusions on precision such as Figure 6 are drawn from, so we have reasonable confidence in the estimator's error and performance for the dimensionality and sample size present.

Similarly, for this dataset if we intentionally increase the max-norm nearest neighbor distances (and thus scale of operation) by choosing k>4 (as seen in supplemental figure S7), we can observe that estimated precision does not change. Higher dimensional muscles did not have precision estimates vary any more or less than the other muscles, with precision estimates for all muscles invariant to the scale of operation determined by k at least as far out as k=7.

To raise this point and cover its investigation to readers of the paper, we have included a version of the above points in the discussion section at L457-465, starting with "One confound to this observation…"

2. Continuous-vs-discrete estimators

Before even reading the results, I'm inclined to believe that the continuous estimator is more reliable than the discrete valued estimator, as per the good points you make in your introduction and as demonstrated for other information-theoretic estimators on spike trains recently e.g. Shorten et al PLOS CB 10.1371/journal.pcbi.1008054.

However I think there are several issues with your comparison here:

*A. You are discretising both the spikes as well as the motor output here. For the spikes you are investigating how fine grained you can make the resolution (which is the make question of the paper), but you're only looking for information to the 2x2 state motor outputs with the discrete estimator. In contrast, the continuous estimator looks for information in the resolution it can work to on *both* the spikes and the motor outputs. You highlight this yourself at lines 264+: "highlighting the importance of within-wing stroke features of the motor output that are not captured in the four motor output state space in the discrete method". However I don't think you recognise this as a confound for your main question of how fine you can make the resolution. In this sense, you may be actually handicapping the NSB estimator for addressing that particular question - if you want to make a fair comparison you would need to allow it to explore other resolutions on the motor outputs as*
well. Otherwise you're simply preventing it identifying information that the continuous estimator has access to.
Because of this, you simply cannot claim the continuous estimator has found more information in the spike times. To address this you would need to either: a. allow the NSB to try more fine grained discretisations of the motor outputs as well, or b. give the KSG estimator the same binarisations of the motor outputs (it is possible to have an adapted KSG estimator exploring MI between continuous data and discrete, see e.g. Ross PLOS One 10.1371/journal.pone.0087357)

This is an important point that we have now altered text and figures throughout the paper to address (such as the paragraph starting at L311, “Part of what hinders the discrete method…”). NSB, for the dataset of this paper, struggles with bias in the small resolution, high bin count regime, a problem which is worsened by finer discretization of motor outputs, particularly for muscles with more than one spike per wing stroke (see the new supplemental figure S4 which explores the performance of NSB with different levels of motor discretization). We have taken the suggestion to test NSB on other discretizations, we have upped the motor output resolution NSB is run at in most of the main text figures to 9 total states, and implemented a shuffling bias correction to alleviate the high bias present in the small resolution regime. As S4 shows, estimated precision from the bias-corrected discrete method does not actually change with increasing resolution of motor output; rather, the limitations of the estimator itself, discrete binning of spike times, and other issues with undersampling bias are likely more at play. The particular passage of text mentioned has been altered to better reflect this point. Thank you for the suggestions that have improved our comparison of the methods.

B. Some of the conclusions in the results on the KSG estimator working better than NSB seem rather odd. Line 238 - "could not detect the precision obtainable by the continuous estimation method, as seen in Moth 1’s L3AX (Fig 4A)". I assume you mean that the NSB method reached its peak at more coarse grained resolution r_d than the peak for KSG at finer r_c. Yet, the KSG method estimates a smaller amount of information than the NSB. This also seems to be especially the case for RDAs. It seems difficult to make a case for better precision when it's measuring less information overall - what does it matter where it cuts off if there is provably more information there than that estimator can identify? I would assume this is impacted by point C below, and addressing that may help here?

Indeed, the difference in magnitude of information was purely due to KSG estimating the conditional MI of spike timing separate from spike count, while the discrete method estimates both. For this study, the total amount of information is not the relevant quantity of interest; instead, the scale at which any information is carried, found as the point at which MI begins to fall with increasing added noise amplitude, is the quantity of interest for spike timing precision. However, though the addition of spike count information to the KSG method does not change the precision estimate, it does change the amount of information shown. To help with clarity and ease of comparison we’ve added spike count information to the KSG method, see our response to point C below.

You go on to say at line 239 - "Still other examples never plateau at high bin sizes" - but you don't tell us why this is a problem. One counter argument might be that the NSB estimator is simply able to keep moving to these finer grained resolutions given the amount of data, and there is in fact no problem. I.e. the true precision may be lower than we're looking at here. I do agree with your motivation in looking for the plateau as indicator of stability before undersampling bias kicks in, but it's not well explained, and perhaps it's just not always there to identify anyway (i.e. in some cases there may be no plateau before the undersampling bias takes off). I would recommend you take a look at the recent article of Rudelt et

al., PLOS CB 10.1371/journal.pcbi.1008927 using an NSB estimator where an optimum dimensionality (akin to the number of bins provided by your resolution here) becomes much easier to identify as a peak rather than a plateau by compensating for bias,
either by Bayesian bias criterion (BBC) or Shuffling bias correction. (Here you would e.g. shuffle the samples of your motor outputs w.r.t. the spikes, and compute the estimates on the shuffled data as the bias to be subtracted out). In other words, the increased dimensionality / number of bins increases the bias, and you're looking for the point at which increasing the number of bins pulls out the maximum information before such increases are dominated by bias.

> We thank the reviewer for this reference and insightful point; the text previously had little to no direct mention of the NSB estimator not reaching a plateau being an indication of undersampling bias. We've added much more reference to this in the text (such as the paragraph at L174-181, "While the NSB entropy estimator typically performs…"), cited the Rudelt paper you referred to (such as at L182), and implemented shuffling bias correction as suggested, converting the discrete method to a peak finding estimation rather than a plateau drop-off estimation. While our overall conclusions did not change, we found it quite helpful in making the discrete method more usable and tractable. Bias correction allowed us to explore higher resolutions of motor output discretization and allowed for estimating the precision for our entire dataset using the discrete method. As the new Figure 4 and supplemental figures S3 and S4 show, the shuffling bias correction method works well at dealing with the undersampling bias issues previously present, and we've incorporated discussion of it and the undersampling bias issues NSB faces at high numbers of bins throughout.

C. You're comparing MI from one estimator with a conditional MI from the other.
- In the first place, the meaning of the sum in (4) is not immediately clear. After some revision, and clues later in the article, I realised that i is the total number of spikes in a wing stroke, so we're calculating MIs separately for wing strokes with the same number of spikes, then taking a weighted average of these, which makes it a conditional MI (conditioned on the number of spikes). I think it makes sense that you've done that, but your text needs to be a whole lot clearer that this is what you're doing. (For a while it looked to me to be summing over the contribution of each of the i spikes).
- More importantly, there are implications then for the discrete-continuous comparison. As per line 271 - "Finally, the KSG MI estimation only includes information encoded by spike timings, whereas the NSB MI estimation includes information about spike rate in its entropies". This wasn't so clear to me initially whilst I was still trying to unroll precisely what equation (4) was measuring as above. Once I realised that it was a conditional MI (conditioned on the number of spikes in the window), this line makes perfect sense. But it thus prompts other questions: aren't we comparing apples with oranges in comparing the MI from one estimator with a conditional MI from the other then? Perhaps this explains why the NSB estimator seems to capture more information in some situations in Fig 4, and Supp figs S2 and S3? Shouldn't we simply compute the MI from the number of spikes to the motor output and add this to the KSG value (using a mixed discrete-continuous estimator as above)
-- this would be better -- or else subtract it from the NSB estimator (using the discrete estimator)? It may help to address the weirdness from having vastly different plateau levels, as per point B above.

Thank you for prompting us to clarify here. While our measurement of spike timing precision is agnostic to the magnitude of information carried, so precision results are not affected by information in spike count, we recognize using only a conditional information for KSG but total information for NSB is a point of confusion. To alleviate this, and allow readers to more directly compare the two methods not just on precision observed but the actual magnitudes of information, we've added spike count information to all information values from the KSG estimator shown in the paper. Examples of this would include Figure 4, and supplemental figure S2.

We've also altered the mentioned equation (now equation 5 at L213) and surrounding text for greater clarity, and to reflect the change to including spike count information.

3. By limiting to two PCs of the motor waveform, are we limiting information that we can measure here? This does not seem to be discussed anywhere. Your previous article shows substantial amounts of information still in the PCs beyond the first two, even though they seem to hold the largest. (That said, the first two PCs are not picked because they hold the most information from the spikes, but because they contain the most variance explained by the spikes or not - what if they were independent of the spikes, but the information was held in other PCs?). The first two components might hold much of the information, but how confident can we be that the conclusions on scales would still hold if we added other components in?

We thank the reviewer for asking this insightful question. It is possible that variation captured in higher principal components would be encoded by spike timings and affect our estimates of precision. However, in our previous article, we present an argument for using the first two principal components that hinges on the trade-off between variation captured and potential bias/instability in our information estimates.

With each PC used in our information estimates, we are obtaining diminishing returns on variance explained (see Fig. 1F in Putney et al. 2019). We also show that the first two PCs in this particular data set capture features of the yaw torque waveforms across different deciles of motor outputs (see Fig. 2 in Putney et al. 2019). In total, the first two PCs capture on average 78% of the variance present in the original data. We did the information estimates with no noise added on three PCs and were able to replicate comparable results to using two PCs (see Fig. S5 in Putney et al. 2019), and for most muscles the information values do not show significant changes with changing numbers of PCs beyond 2 (see Fig. S2-S3).

However, including more of the original variation in the motor output would be ideal, but with increasing dimensionality of our motor output we begin to trade off stability in our mutual information estimates. HIgher dimensionality means we would need more data observations, and could introduce bias given our limited sampling in experimental data sets. When we did our analysis with 3 PCs, we saw individual muscles that had poor behavior in information estimates across different values of k, the number of nearest neighbors, and sub-sampling into data fractions. This problem would only be worse in the NSB method, where adding another principal

component would increase the number of motor output states to 8 even if data is only divided into high and low states on each principal component axis.

Therefore, we chose to use 2 PCs in this paper because it captures the majority of the variation in the motor output while not sacrificing the quality of our mutual information estimates. Additionally, we wanted to be able to make the MI estimates we did with noise added comparable to our previous results.

We acknowledge that this discussion would make a valuable addition to the paper, so have made additions to the text at L104-110 to address this question.

Minor points:
- line 205 or so - it would be good to be a little more clear on the interplay between using 150 different additions of noise, and the sub-sampling to come up with the standard deviation of estimates. I think for each sub-sampling we average over the 150 different noise additions to obtain an average MI, then we have a population of these average MIs that we determine the standard deviation across?

This is correct. The sub-sampling only applies to the true MI estimate, where no noise is added. This was done previously for this data set based on the technique discussed in Holmes et al. 2019 (which is cited). However, we realize this language may have been confusing and have updated it to clarify on L229-239.

- fig 4 - Left column label LAX -> L3AX ?

Corrected.

- fig 5 caption - should probably point out that spike rates are relative to DLM here (as the main text does later)

We apologize for any confusion we may have introduced, but the DLM spikes once per wing stroke, and all the spike rate values presented here are absolute spike rates, not relative to the DLM. If the reviewer meant spike times, instead of spike rates, those are also not relative to the DLM, but instead relative to the start of the wingstroke found using the Hilbert transform of z-force. This process we have made sure to clarify at lines L110-L115.

- what are the actual values for L and N in this data set?

These values varied in each individual moth, since we had different numbers of wing strokes for each moth and each individual varied in the length of its shortest wing stroke. We have reported the range of values for N and L across all of the seven individuals in the caption of Figure 1.

- line 78 "spike timing precision may be smaller" -> finer?

We have changed this language in response to another reviewer, and thank the reviewer for pointing out the potential confusion of using “small” to describe precision.

- equation (4) - need consistent use of ; in MI notation - the first two just use a comma.

Corrected.

- Your KSG estimates are quoted in bits - just wanted to double check that's definitely what you've measured them in. It's definitely better for comparison to the discrete estimates, but since the digamma values give values directly in nats rather than bits this is worth double checking.

We thank the reviewer for double checking our units. We are reporting in bits, and have modified the output from both the KSG and NSB estimators to convert from nats to bits.
